# Novel Spatio-Temporal Continuous Sign Language Recognition Using an Attentive Multi-Feature Network

**DOI:** 10.3390/s22176452

**Published:** 2022-08-26

**Authors:** Wisnu Aditya, Timothy K. Shih, Tipajin Thaipisutikul, Arda Satata Fitriajie, Munkhjargal Gochoo, Fitri Utaminingrum, Chih-Yang Lin

**Affiliations:** 1 Department of Computer Science and Information Engineering, National Central University, Taoyuan City 32001, Taiwan; 2 Faculty of Information and Communication Technology, Mahidol University, Nakhon Pathom 73170, Thailand; 3 Department of Computer Science and Software Engineering, United Arab Emirates University, Al Ain 15551, United Arab Emirates; 4 Faculty of Computer Science, Brawijaya University, Malang 65145, Indonesia; 5 Department of Electrical Engineering, Yuan-Ze University, Taoyuan City 32003, Taiwan

**Keywords:** continuous sign language, spatial, temporal, multi-feature, keypoints, self-attention

## Abstract

Given video streams, we aim to correctly detect unsegmented signs related to continuous sign language recognition (CSLR). Despite the increase in proposed deep learning methods in this area, most of them mainly focus on using only an RGB feature, either the full-frame image or details of hands and face. The scarcity of information for the CSLR training process heavily constrains the capability to learn multiple features using the video input frames. Moreover, exploiting all frames in a video for the CSLR task could lead to suboptimal performance since each frame contains a different level of information, including main features in the inferencing of noise. Therefore, we propose novel spatio-temporal continuous sign language recognition using the attentive multi-feature network to enhance CSLR by providing extra keypoint features. In addition, we exploit the attention layer in the spatial and temporal modules to simultaneously emphasize multiple important features. Experimental results from both CSLR datasets demonstrate that the proposed method achieves superior performance in comparison with current state-of-the-art methods by 0.76 and 20.56 for the WER score on CSL and PHOENIX datasets, respectively.

## 1. Introduction

Sign Language prioritizes manual communication using hand gestures, body language, and lip movements instead of sound to communicate [1,2]. Usually, sign language is used by people who are deaf or hard of hearing, but it can also be used in situations where it is impossible or difficult to hear sounds. Therefore, a sign language recognition (SLR) system is needed since it helps to connect people who are hard of hearing and those who are not. 

In recent years, researchers have focused much attention on SLR because of the rich visual information it provides. Recent SLR studies is usually grouped into isolated sign language recognition (ISLR) or continuous sign language recognition (CSLR). Several works address only ISLR [3,4], while others only analyze easier tasks, such as static gestures for alphabet recognition [5]. Meanwhile, the latest methods are usually more complicated as they solve CSLR tasks [6,7,8]. Compared to ISLR, CSLR is a more challenging problem as it involves the reconstruction of sentences. 

CSLR research is still in great demand because its implementation is closely related to everyday conditions in the real world. The aim of this approach is to recognize the series of glosses that occur in a video series without clear segmentation or even none at all. Furthermore, it incorporates a great deal of machine learning research and a thorough understanding of human behavior. For instance, it involves human movement tracking [9], gesture recognition [10], and facial recognition [11]. Nevertheless, there are several challenges to performing CSLR tasks.

First, data collection and annotation are expensive for CSLR [12]. This is perhaps one of the challenges faced in its development, since the CSLR involved in a large network and the amount of data strongly affect the performance [13]. Moreover, several available datasets for sign language are weakly annotated [12,14,15]. In order to solve this issue, numerous studies have used a weakly supervised approach, alongside application of an alignment and a feature extractor module to the network architecture [12].

Second, compared to ISLR, CSLR is more complicated. Sufficient information is acquired by using several features; this has been proven to achieve better performance than using a single feature as reported in previous works [16,17,18]. These multiple features consist of the main feature which is a body image that achieves the highest accuracy and additional features, such as pose, head, left hand, and right hand, which has lower accuracy for individual performance [17,18]. Training a large network with a large amount of data is time consuming [13]. Adding the input stream also increases the training time, while using additional image-based features increases the cost [19]. Therefore, we need to choose important features so we can train efficiently.

Third, video input has a large number of images in the sequence. Some images have an unclear hand shape due to the fast movement, possibly leading to incorrect information. Therefore, our proposed model utilizes self-attention based on [20] to help select important information. Moreover, self-attention proven by [21,22] has an impact on enhancing performance.

Therefore, we propose a novel model called the novel spatio-temporal attentive multi-feature (STAMF) to handle all problems. We followed previous works [17,23], which has been proven to work for CSLR with weak annotation problems. They construct the model using three main components: first is the spatial module, second is the temporal module, and third is the sequence learning module. We propose efficient and effective multi-feature input using the full frame feature along with keypoint features to perform CSLR tasks. The full-frame feature represents the body image as the main feature, and the keypoint features as the additional feature. The keypoint features is the body pose, including the detail of the hand pose. This body pose is the most effective additional feature since in some works it has been proven to achieve the highest accuracy after the full-frame feature [17,18]. We also utilize an attention module that uses self-attention based on [20] to capture the important feature and to help the sequence learning to enhance performance.

The contribution of this manuscript is summarized as follows:

- We introduce novel temporal attention into the sequence module to capture the important time points that contribute to the final output;
- We introduce the multi-feature that consists of the full-frame feature from the RGB value of the frame as the main feature and keypoint features that includes the body pose with the hand shape detail as an additional feature to enhance model recognition performance;
- We use the WER metric to show that our proposed STAMF model outperforms state-of-the-art models on both CSLR benchmark datasets through the experiments.

## 2. Related Works

There have been several advancements in technology, and a lot of research has been done for SLR. Previous studies [24,25,26,27] explored the possibility of using ISLR that have a segmentation for each word. In recent years, deep learning-based methods have been used to extract features using convolutional networks, either 2D [28,29] or 3D [30,31], for their strong visual representation. The majority of early research on sign language recognition centered on ISLR with multimodal characteristics [30,31,32], such as RGB, depth maps and skeletons, which give a better performance.

Nowadays, CSLR has become more popular, although it has not been segmented clearly between each word. Early works use a CNN feature extractor [6,33] and HMM [34] to build the sequence target. Some recent research for CSLR systems [17,23] has included three main steps in performing the task of problem recognition. First, they conducted the spatial feature extraction, then temporal segmentation, and finally sentence synthesis with a language model [35], or they used sequence learning [17,23]. This sequence learning used Bi-LSTM and CTC to mine the relationship between sign gloss in the video sequences. Even though it uses a weak annotation that has unsegmented video sequences to define the sign glosses, these approaches have shown promising results.

However, the most recent related CLSR study that implemented a multi-feature approach [17] used five features simultaneously. The multi-feature approach is heavier compared to using fewer features [19]. This approach also cannot handle the noisy frames from the video sequence that have unclear information, such us a blurry hand shape due to fast movement. Moreover, relying on RNN based sequence learning may encounter problems with long sequence and may lose the global context [20].

The current research aims to improve performance by adding a self-attention mechanism [21,22] that can handle longer sequence to learn the global context. Self-attention is based on early research [20] that showed that self-attention has the advantage of being able to handle long dependencies. However, this self-attention is easier to learn a shorter path compared to a longer path in long dependencies. In the previous CLSR works [21,22] self-attention could help the network to learn the feature more effectively.

Therefore, in this paper we introduce a novel spatio-temporal attentive multi-feature model. This proposed model effectively extracts the important features and learns the sequence better by giving important information using a self-attention mechanism from multi-feature. All the processes are executed in an end-to-end approach.

## 3. Proposed Method

This section details the core techniques of our proposed model for CSLR. Therefore, we begin this section by explaining our proposed model’s overview. In addition, we provide more details about each key component, including the spatial module, the temporal module, and the sequence learning module. In addition, we also explain our proposed attention module to help the model learn better. Finally, we can integrate the framework for training and inferencing into our proposed model.

### 3.1. Framework Overview

**Figure 1:**
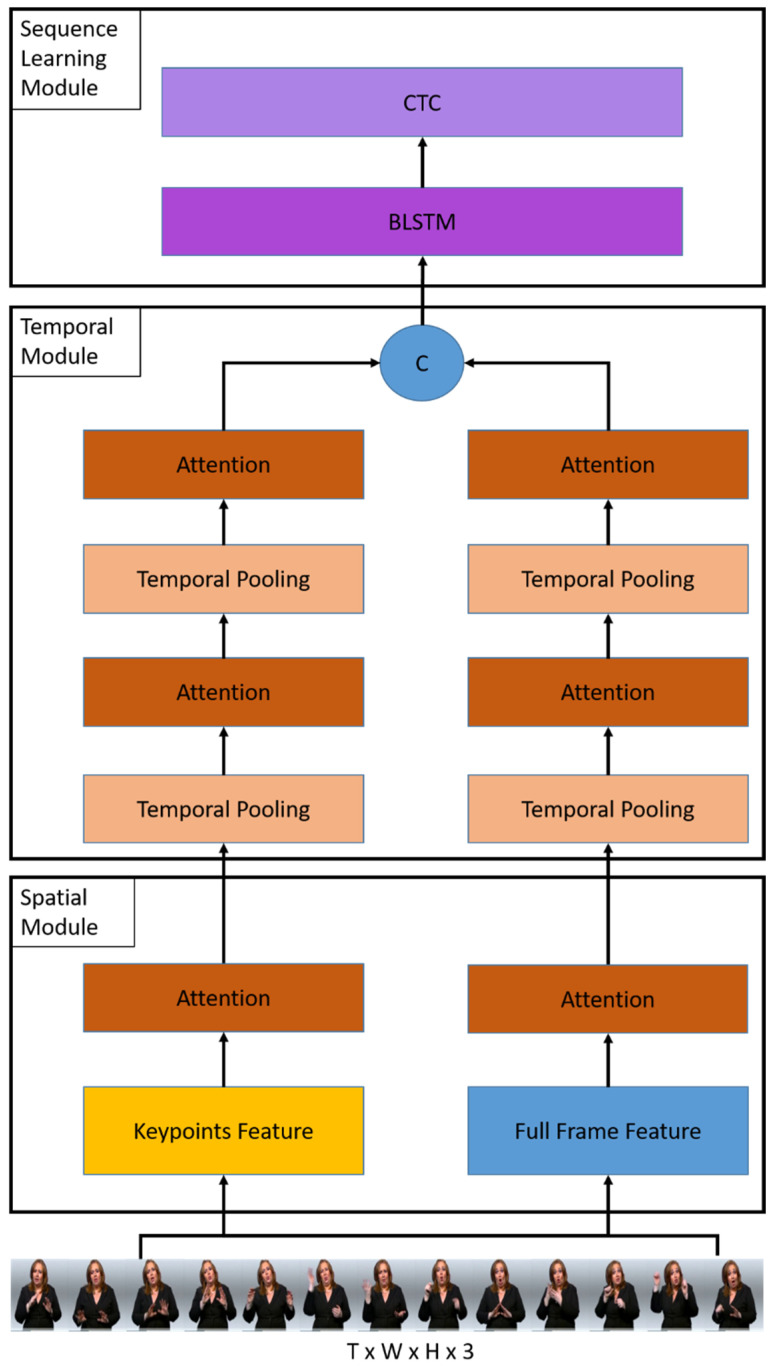
The overall architecture of the proposed method consists of three components: a spatial module, a temporal module, and a sequence learning module. The spatial module first takes the image sequence to extract frame-wise features and then applies the temporal module to extract the temporal features. Then, the temporal features are sent to the sequence learning module to perform word prediction and construct it into a sentence.

Given a video input, our proposed model aims to predict the corresponding sign into a correct gloss sentence. The first module generates multiple spatial features, such as full-frame and keypoint features for each *T* frame of the video. Then, the temporal module allows us to extract temporal correlations of the spatial features between frames for both streams. As a final step, the spatial and temporal networks have been linked to bidirectional long-short term memory (Bi-LSTM) and CTC for sequence learning and inferencing. Next, we explain our main components in more detail and consecutively. The overview of our proposed architecture is shown in Figure 1.

### 3.2. Spatial Module

**Figure 2:**
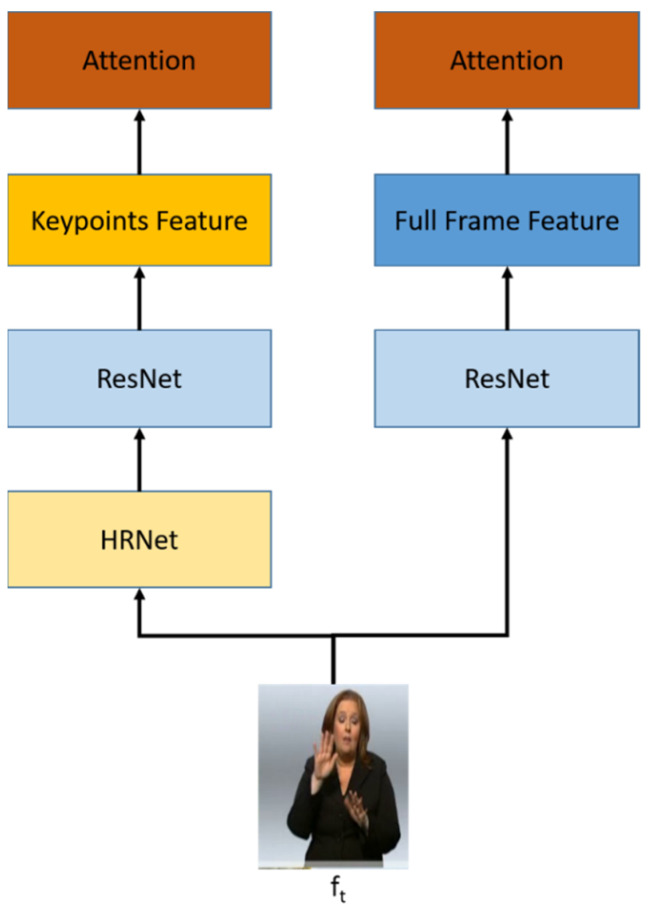
The spatial module architecture uses multi-stream input. RGB stream as a full-frame feature and keypoints stream as a keypoint features.

The spatial module exploits a full-frame feature and keypoint features, as shown in Figure 2. This module uses 2D-CNN network architecture as the backbone, and ResNet50 is chosen to capture the multi-features. ResNet50 is more effective to be used compared to recent ResNet architecture in terms of time, while having a comparable result [36,37]. The RGB uses ResNet50 directly, while keypoint is obtained by HRNet [38] from the video frame and is extracted using ResNet50 to get the keypoint features.

#### 3.2.1. Full-Frame Feature

**Figure 3:**
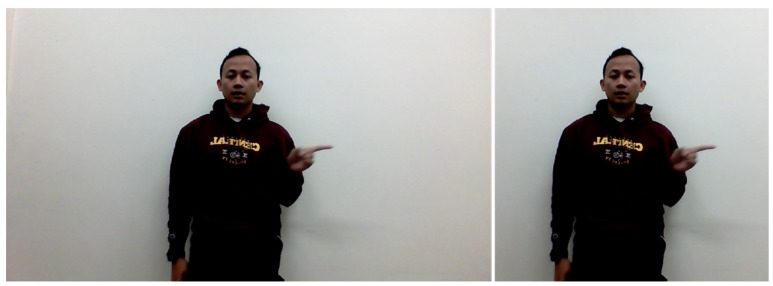
Full-frame feature using RGB image, the (**left** image) is the original image, and the (**right** image) is the cropped image to adjust with the proposed model.

We applied our preprocessing steps to the RGB data then fed our data into the model. We then put them as a full-frame input into our architecture. Figure 3 shows the illustration of the original RGB image at the left side and the cropped image at the right side. The cropped image used as input by the model. This illustrates the preprocessing step that reduces the less important parts of the image and puts more focus on the signer. This cropping uses random cropping method from [12] to augment the dataset. The full-frame feature is extracted from the cropped imaged for each frame in the sequence using the ResNet50.

#### 3.2.2. Keypoint Features

**Figure 4:**
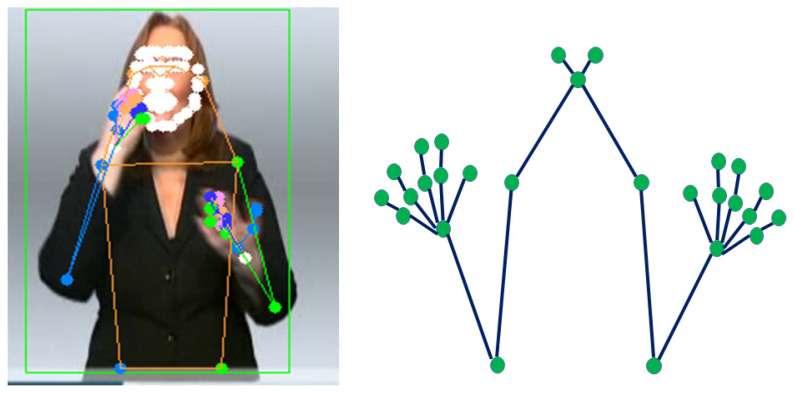
Keypoint features of PHOENIX-RWTH dataset [33,39], (**left** image) extraction from RGB image, and the (**right** image) is the selected keypoint used by the proposed model.

We extracted the keypoint features in the spatial module from the data RGB for each frame in the video input. The quality of keypoint features has an important role in our proposed model, so we need to use a robust approach, such as HRNet [38]. We employed pretrained HRNet [38] to estimate all of the 133 body keypoints, and we utilized 27 out of the 133 keypoints from its result. As shown in Figure 4, the left side is the original upper body keypoint, and the right side is the selected 27 upper body keypoints. These 27 keypoints include wrists, elbows, shoulders, neck, hands, and fingers.

### 3.3. Temporal Module

**Figure 5:**
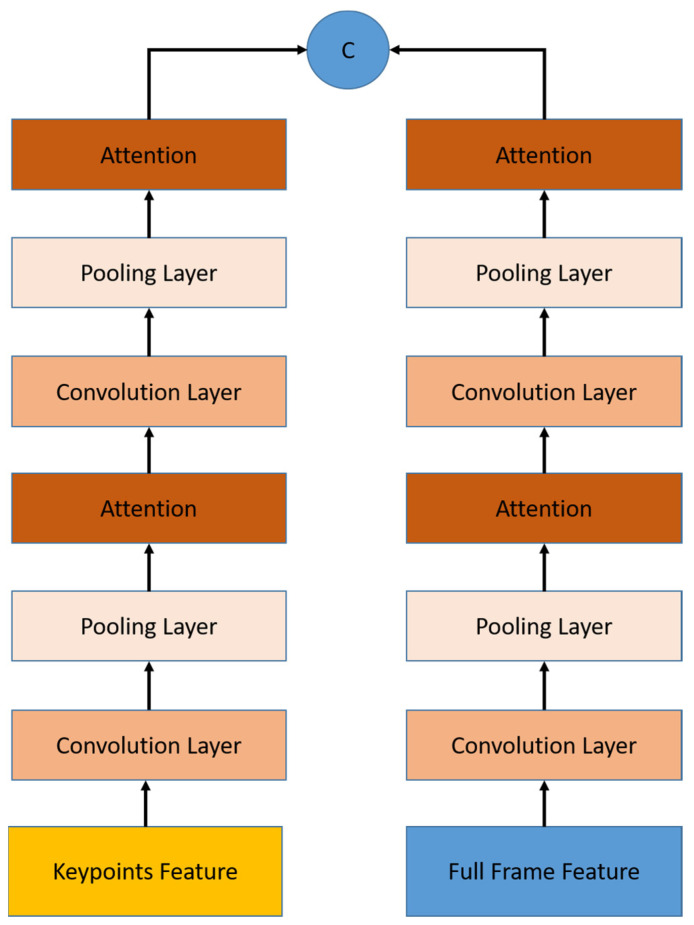
Temporal module architecture consists of a stacked 1D-CNN and pooling layer embedded with attention module. Work in parallel for both feature streams, concatenated at the end of the stacked layers, and produce a single temporal feature with a sequence length four times smaller.

The temporal module aims to learn spatio-temporal information from the spatial module. Temporal modules are constructed by stacked Temporal Pooling for each stream. As shown in Figure 5, the Temporal pooling module consists of a temporal convolution layer and a pooling layer to extract features from sequential inputs.

The input is a list of spatial multi-features from the previous stage. The temporal feature is obtained using the temporal convolution layer which is a single 1D convolutional layer with the same input and output lengths, followed by a single pooling layer that decreases the size to a half. Using these two stacked temporal pooling layers is the best configuration, according to the previous works [12]. After each temporal pooling, we embed an attention module that will be explained in detail in Section 3.4. At the end, we concatenate the output of temporal pooling from both streams.

### 3.4. Attention Module

**Figure 6:**
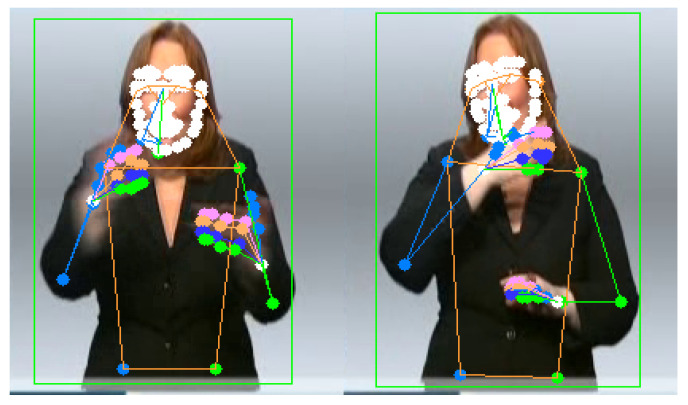
Illustration of defect frames on RWTH-PHOENIX dataset [33,39]. Some of the keypoints in the hand area are in the wrong position due to blurry images.

The video has multiple frames where some parts of the image are sometimes blurry. The RTWH-PHOENIX dataset [33,39] has more defective frames than the CSL dataset [8,40,41]. This happens when the movement is too fast, creating a blurry image and resulting in the wrong keypoint location. This frame is considered defective and potentially leads to misinterpretation of both the RGB and keypoint features. Figure 6 shows an illustration of defective frames in the RTWH-PHOENIX dataset [33]. In order to deal with this problem, we added an attention layer.

Using the CTC algorithm, alignment of the path along with its labeling is performed by using a blank label and removing the repeat labels. CTC prefers to predict blank labels rather than gloss boundaries when it cannot distinguish the gloss boundary, but none of the results are convincing. This leads the network to use CTC to produce spikes in results when analyzing, learning, and predicting [42,43]. Generally, the CTC loss seeks the keyframes, and the last result is the prediction of a particular keyframe that has a high probability of being a blank label or a nonblank label. If the gloss predicts the same label or blank label consecutively, it results in the same output. However, if there is an insertion label in between the same label, even if there is only one mistake, it results in a much bigger loss. Here the addition of an attention layer helps to select the important temporal sequence before being used for sequential learning.

The attention module uses a multi head self-attention mechanism [20]. The multi-head module is used to run several parallel attention mechanisms at the same time. Multi-head attention runs independently to focus on the short-term dependencies or the long-term dependencies in a separate head. Each output is then concatenated linearly and transformed into the desired shape.

Concurrently, the multi-head self-attention mechanism takes care of information from multiple representation subspaces, depending on the history of observations. For simplicity, we denote the input sequences as X. Mathematically, for the single-head attention model, given input *X*^*t* − *T* + 1:*t*^ = [*X*^*t* − *T* + 1^, · · ·, *X*^*t*^ ] ∈ ℝ^*T* × *N* × *P*^, three subspaces are obtained, namely, the query subspace *Q* ∈ ℝ^*N* ×*dq*^, key subspace *K* ∈ ℝ^*N* × *dk*^, and the value subspace *V *∈ ℝ^*N* × *dv*^. The latent subspace learning process can be formulated as [20]:

*Q* = *XW^Q^*, *K* = *XW^K^*, *V* = *XW^V^*, (1)

Then, the scaled dot-product attention is used to calculate the attention output as [20]:

(2) $$Attention(Q,K,V)=softmax(QK^{T}/\sqrt{d_{k}})V,$$

Furthermore, if we have multiple heads that concurrently follow the multiple representations of the input, we can obtain more relevant results at the same time. The final step is to concatenate all of the heads and project them again to calculate the final score [20]:

*MultiHead*(*Q*,*K*,*V*) = *Concat*(*head*_1_,..., *head_h_*)*W^O^*, (3)

*head_i_* = *Attention*(*Qi*,*Ki*,*Vi*), (4)

where *Qi* = *XW^Q^_i_*, *K_i_* = *XW^V_i_^*, and *W^O^* ∈ *R^hd^* × d_model_. Finally, it can select the important part from sequence of features because not all information in the sequence are important.

**Figure 7:**
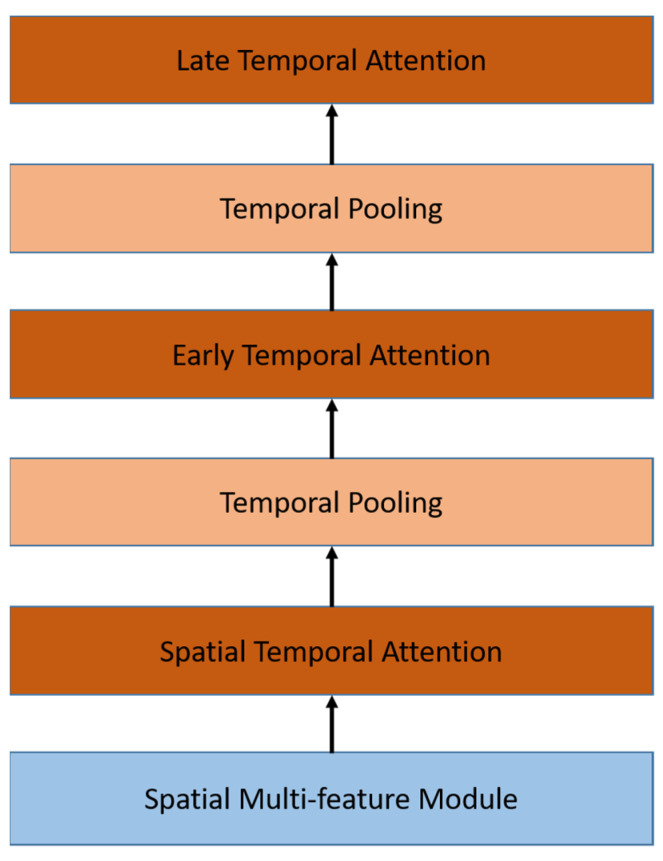
Attention modules are embedded in spatial and temporal modules in different configurations.

As shown in Figure 7, we use the attention module in several configurations. The first attention module is placed in the end of the spatial module, while the second and third attention modules are placed in the temporal module. The second attention module, called the early temporal module, is placed after the first block of temporal pooling as input, whereas the third temporal attention module, called the late temporal attention module, is placed after the second block of temporal pooling.

### 3.5. Sequence Learning

After getting the results from the spatial and temporal feature extractor in the form of a gloss feature, we need to arrange them into a complete sentence. Therefore, we need to use sequence learning to ensure that the gloss is managed in order to form a good sentence. The most prevalent method in the current research refers to Bidirectional Long Short Term Memory (Bi-LSTM) and Connectionist temporal classification (CTC) [17,23] to determine the greatest probabilities from all potential alignments. There are many sequence problems that can be solved using CTC and several recent works [17,23] propose CTC as an end-to-end training process for CSLR.

Long short-term memory (LSTM) [44] is a variant of the recurrent neural network (RNN), and it is widely used in sequence modeling. LSTM excellently models long-term dependencies; it can process entire sequence of inputs and use their internal state to model the state transitions. However, the shortcoming of this forward RNNs is that the hidden states are only learnt from a one-way direction. The RNN produces a hidden state as described by the equation below after receiving the feature sequence as input.

*h_t_* = RNN(*h_t_*_−1_,*o_t_*), (5)

where *h_t_* represents the hidden state, where the initial state *h*_0_ is a fixed all-zero vector, and *t* is the time step. RNN is used to predict the sign glosses according to the spatial feature sequence input.

Moreover, SL tasks are quite complex. Therefore, it not only requires features received from a one-way context but also needs assistance with the use of two-way information to learn the occurrence of words that come before and after the other words in the sentence. Consequently, we utilize Bi-LSTM [45] to learn the complex dynamic dependencies of image sequences by transforming them using a spatial and temporal representations into sequences of gloss features. According to the explanation stated earlier, the Bi-LSTM method is able to perform two-way work with the LSTM method. This means that the Bi-LSTM method can better classify sequential data. The computation of Bi-LSTM that uses two-way hidden states can also be seen as repeated computations of the LSTM that is processed from front to back and then from back to front. Afterwards, the hidden state of each time step is passed through a fully-connected layer and a softmax layer [17].

a*_t_* = *W(ht + b)*, (6)

(7) $$y_{t,j}=\frac{e^{a_{t,j}}}{\sum_{k}e^{a_{t,j}}},$$

where *b* is a bias vector and *y*_(*t*,*j*)_ represents the probability of label *j* at time step *t*. In our TSL task, label *j* is the vocabulary that is obtained from the sentence. In practice, Bi-LSTM significantly improves the amount of information a network can access, which in turn improves the context of information that is available in the algorithm. The information contains the knowledge of which word comes after or before the current frame in the sentence feature sequence input.

Bi-LSTM sends hidden states as output to the CTC layer for each time step. Since the results of this Bi-LSTM do not pay attention to the gloss arrangement, we use CTC to handle video sequence mapping $o={\left\{o_{t}\right\}}_{t=1}^{T^{\prime }}$ to produce a sign gloss sequence $\ell ={\left\{\ell_{i}\right\}}_{i=1}^{L}$ (*L*≤T) with better arrangement. CTC is used in many fields, including hand writing recognition [46], speech recognition [47], and sign language recognition [17,23]. In this domain, it is used as a scoring function without aligning the input and output sequences. In CSLR, CTC is known as a module developed for end-to-end tasks involving the classification of temporal data without segmentation. Through dynamic programming, CSLR is able to solve the alignment issue by creating a blank label (-) which permits the system to produce optimal results for the representation of data that does not have labels (such as epenthesis data and segments of non-gesture data). In particular, CTC generates a blank label “-“ to extend the vocabulary *V*, where *V* = *V_origin_* ∪ {-}. The purpose of this blank label is to represent the transition and to notify the existence of a blank gloss that does not provide information for the learning process, as $\;\pi ={\left\{\pi_{t}\right\}}_{t=1}^{T^{\prime }}$, where $\pi_{t}\in V$. As a result, CTC is able to manage the output parameters from the spatial and temporal feature extractor and also the Bi-LSTM as the alignment module by summarizing the probabilities of all possible paths. All of the alignment path π have a probability that is given the input sequence as follows [17]:

(8) $$p\left(\pi |o\right)=\overset{T^{\prime }}{\underset{t=1}{\prod }}p\left(\pi_{t}|o\right)=\overset{T^{\prime }}{\underset{t=1}{\prod }}y_{t,\pi_{t}}$$

In the next step, we define a many-to-one mapping operation B, which removes any blank space and duplicates words from the alignment path. For example, *B* (II-am- -a- -doctor) = I, am, a, doctor. As a result, we are able to calculate the conditional probability of the sign gloss sequence l as the total of the probabilities of all routes that can map to *l* from *B*, in the way described below [17]:

(9) $$p\left(\pi |o\right)=\underset{\pi \in {ℬ}^{-1}}{\sum }p\left(\pi |o\right)$$

where *B*^−1^(*l*) = {π|*B*(π) = *l*} is the inverse operation of *B*. Finally, the CTC losses of feature sequence are defined as follows [17]:

(10) $${ℒ}_{ctc}=-\ln p\left(\ell \;|o\right)$$

The conditional probability *p*(π|*X*) can be calculated according to the conditional independence assumption.

## 4. Experimental Results and Discussion

In this section, we will explain the details of our experiment with the proposed configuration as explained in the earlier section.

### 4.1. Datasets

In this section, we explain the datasets that we utilized during this experiment. We used two datasets, first is the CSL dataset [8,40,41] and second is the RWTH-PHOENIX dataset [33,39]. Both of these datasets are continuous sign language datasets, which are used to translate some series of gestures to a full sentence.

#### 4.1.1. CSL Dataset

The CSL dataset has been used by several works [8,40,41]. There are a total of 100 sentences and 178 words present in this dataset, which are commonly used in daily communication. This dataset contains 5 words on average per sentence. Each sentence was performed by 50 signers 5 times. Thus, the total number of videos is 25,000, making this one of the largest datasets. In order to train our CSL model, we divided the dataset into data for training with 20,000 videos and for testing with 5000 videos of the same sentence but with different signers.

#### 4.1.2. RWTH-PHOENIX Dataset

The RWTH-PHOENIX dataset [33,39] is a German sign language dataset, which is a recording of public weather broadcasts from television stations in Germany. This video is processed so that it has a size of 210 × 260. There are 6841 different sentences signed by 9 different signers. All of the signers wore dark colored clothes on a light colored background. In total, there are 1232 words with about 80,000 glosses. This dataset is divided according to a predetermined format, consisting of 5672 training samples, 540 validation/dev samples, and 629 test samples.

### 4.2. Data Preprocessing

The first thing we needed to do was preprocess our dataset to fit our proposed model. We performed resizing and cropping, as illustrated by Figure 3; in the left side we can see that the original data was sized at full HD or 1920 × 1080 resolution. We needed to crop and resize it to 224 × 224 resolution since our spatial module receives input that is the same size as the ResNet50 input. After that, we fed the data into our spatial model, which would produce the 7 × 7 tensor from a single image.

The preprocessed full-frame image was then used to generate the keypoint features. We utilized the HRNet model to predict the 133 keypoints from these RGB images. However, we only use 27 keypoint of upper body in our proposed model. The input size for the keypoint features is 27 × 3 since we have 3 axis (x, y, and z) coordinate data per point. The input dimension of the model is *N* × 224 × 224 × 3 for the full-frame feature and *N* × 1 × 27 × 3 for the keypoint input, where *N* is the number of frames. To enhance the variation of the dataset, we follow the augmentation from [12], including random cropping, horizontal flip, and random temporal scaling. Random cropping is also used as part of a preprocessing step to crop the original image.

### 4.3. Evaluation Metric

To evaluate the recognition performance of our proposed model, we use a word error rate (WER) metric, which is a metric commonly used in CSLR to estimate the recognition accuracy of a predicted sentence, as shown by the equation below [17]:

(11) $$WER=\frac{S+I+D}{T}=\frac{S+I+D}{S+C+D}$$

where *S* is the number of substitutions of the original word, *I* is the number of insertions that are not in the original sentence, *D* is the number of deletions that should have been in the original sentence, *C* is the number of correct words that match the original sentence, and *T* is the total number of words in the sentence or which can be obtained from the total of substitutions, deletions, and correct words. For CSL, each character is used to represent a word.

### 4.4. Experiment on Input Streams

This experiment mainly focuses on comparing single stream and multi-stream input on our model. The aim is to find the best configuration of the input stream. We use only the RWTH-PHOENIX dataset in this experiment. Our model would receive two separate inputs, full-frame and keypoint input. The single-stream input uses only the RGB feature from the full-frame image. There are two single-stream input experiments. The first uses only the full-frame feature from the RGB image, and the second uses keypoint features from the keypoints input. The multi-stream input that we propose consists of RGB and keypoint features. The comparison result using a single stream and multi-stream is shown in Table 1. Here, we can see that by using the multi-stream feature we can obtain a better result than by using a single stream. The main stream is obtained from a full-frame RGB feature and the additional stream uses the keypoint features. The additional keypoint stream helps the model to learn better, especially to capture the details of the hand shape.

### 4.5. Experiment on the Attention Module

The aim of this experiment was to choose the best configuration of the attention module. In this experiment, we use the configuration of the best result from the previous experiment result and the same dataset. We denote spatial attention as the model with a self-attention layer at the end of the spatial module for each feature. Early temporal attention is the self-attention layer after the first temporal pooling, and late temporal attention is the self-attention layer after the second temporal pooling. Here, we will compare all of the configurations above and the combination of spatial and temporal attention. The result is shown in Table 2. The result of spatial attention is worse than temporal attention. The spatial level contains a sequence of features for each frame. As mentioned in [20], the self-attention layer is easier to learn the shorter path between long dependencies. Therefore, spatial attention fails to decrease the WER due to the long sequence. On the other hand, we were able to get an improved result using temporal attention by putting it after the pooling so it would get a shorter sequence length. The late attention that has the shortest sequence length obtains the best result. Moreover, the combination of the attention module in spatial and temporal is worse than using only a single attention. Based on these results, we apply the best configuration for the experiments onward.

### 4.6. Ablation Experiment on the STAMF Network

Furthermore, we perform an ablation experiment using the same dataset and the best configuration of input stream and attention module. Table 3 on an experiment using late temporal attention without pooling proves that sequence length affects attention performance. The experiments that use pooling layers with shorter sequence lengths obtain better results. We also perform an ablation experiment to know the performance using different models for sequence learning. The result in Table 4 shows that using LSTM, which has only one-way information, has a lower performance than using Bi-LSTM, which has two-way information.

### 4.7. Experiment on the STAMF Network

In this section, we explain the full training process for our proposed model called spatio-temporal attentive multi-feature (STAMF). This will cover how we fit our input data to the spatial module, temporal module, and sequence learning module step by step. In this section, we use multi-stream input and late temporal attention configuration.

#### 4.7.1. Implementation Details

We perform end-to-end training and testing using input frames with size 224 × 224. For the optimizer, we use an Adam optimizer with 10^−4^ as the learning rate and divide it by 2 at epochs 10 and 15 for the CSL and epochs 30, 40, and 60 for the RWTH PHOENIX. We set the batch size to 4 and perform 80 epochs for the RWTH-PHOENIX and 20 epochs for CSL. The training and testing was run on NVIDIA Tesla V100 and 128G RAM.

At first, we extracted the feature from RGB streams using ResNet50 and used HRNet to obtain the keypoint and then extract the keypoint features using ResNet50. Note that we were dealing with sequential data or a video. Hence, we needed to configure our input size depending on the length of the video. For technical reasons, the input size for all data needed to be identical to each other. Therefore, we must know the longest video in our dataset, then we padded our input frame length to match the largest frame number.

These sequential extracted features were used by the temporal module. Each sequential stream went through two stacked temporal pooling. Each temporal pooling consisted of a 1-dimensional of convolutional network and a max pooling layer, which decreased the length of the sequential by half. The output of the temporal pooling from both streams was then connected to the attention layer and for the last temporal pooling, after the attention layer, it was concatenated at the end as the temporal module output.

The temporal output was processed by the sequence learning module. The process used the Bi-LSTM layer connected with the CTC to get the final alignment and compose the gloss into a final sentence.

#### 4.7.2. Quantitative Result

The final sentences were compared with the ground truth to calculate the WER score as a quantitative result. For the CSL we split the dataset into 80% for the training data and 20% for the testing data. As shown in Table 5, our proposed multi-feature model (STMF) using the full-frame and the keypoint features can achieve a better result and decrease the WER to 0.8 compared to the model using only the full-frame feature [23]. Our best result was obtained by the proposed multi-feature model using the attention mechanism (STAMF), which achieved 0.7 for the WER. The attention layer still slightly improved the result even though the CSL dataset had no defective frames. This proves that the attention layer has an impact on the model. The proposed multi-feature model itself is superior to the state-of-the-art methods and the attention layer contributes to decreasing the WER score on the CSL dataset. The keypoint on CSL gives a significant impact because it can give effective information compared to the other multi-feature method [17] which employs hands, face, and body pose.

For the RWTH-PHOENIX dataset, we used the official configuration to split the training and testing data. The dataset was divided into 5672 sample videos for training, 540 sample videos for self-validation, and 629 sample videos for testing. As shown in Table 6, our proposed multi-feature model (STMF) result using the full-frame and the keypoint features had 24% WER, and our best result was 20.5%, obtained by the proposed model using the attention module (STAMF) in the training result. The test results are second best compared to [17] since they use more features as input. However, the attention module proven makes a significant contribution to decreasing the WER score for the RWTH-PHOENIX dataset. Different from our result for the CSL dataset, our proposed model with multi-stream did not achieve an optimum result. This is because the RWTH-PHOENIX dataset has many defective frames; they have a blurry part, especially in the hand area. This frame gives inconsistent keypoint information due to the missing or incorrect keypoint position and makes the model less optimal. This problem is handled by adding the attention layer to our proposed multi-feature model, which helps to select the correct information and results in significant improvement compared to the proposed multi-feature model without attention.

#### 4.7.3. Qualitative Result

**Figure 8:**
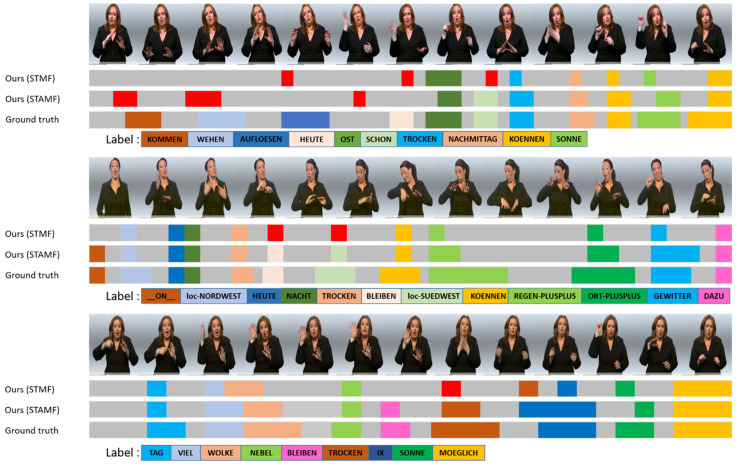
Qualitative evaluation of the recognition result using a different configuration of the RWTH-PHOENIX dataset [33,39]. The wrong predicted glosses are marked in red.

We also performed a qualitative evaluation of our model, using a visualization of the recognition result. Figure 8 shows the details of our qualitative evaluation using three samples of sentences. The first sentence consists of 10 words and the total number of frames is 220. The second sentence consists of 12 words and the total number of frames is 168. The third sentence consists of 9 words and the total number of frames is 135.

The sample result shows that some glosses are not correctly predicted by the models, and we denote it with the red mark. However, it can still predict the majority of correct words. The greatest number of errors is in the first sentence, which has the longest frame number. In this sentence, the beginning of the sentence has more errors, due to several gestures for the early words being only slightly different so it is difficult to distinguish the boundaries. However, the proposed model with multi-feature and attention (STAMF) could identify more words, but they were mislabeled. Moreover, the model configuration with attention achieves a better recognition result for another two samples, compared to the proposed model without attention.

## 5. Conclusions

In this paper, we present a novel spatio-temporal attentive multi feature (STAMF) for CSLR, which aims to learn spatial-temporal correlations and the important information from the sequence of visual features and keypoint features in the end-to-end approach. In our framework, a spatial module was developed with a full-frame feature from RGB data and keypoints from body keypoints, including the hands as multi-features. These combinations used as multi-stream feature input gave superior performance compared to the state-of-the-art approach that uses only full-frame or compared to the method that uses another multi-feature combination, especially for the CSL dataset. Then, we propose a temporal module composed of the temporal pooling and temporal attention to capture the important information in the temporal domain. The addition of late temporal attention is capable of obtaining the important feature from the sequence that has incorrect information and gives significant improvement compared to our proposed multi-feature model. It also helps the sequence learning to learn better and enhances the performance as in the experiment of the CSL dataset. Extensive experiments on commonly used datasets demonstrate the superiority of our proposed model over the state-of-the-art model with WER scores decreasing by more than 50% for the CSL data set and decreasing by 5% for the RWTH-PHOENIX dataset. In the future, we plan to implement the transfer learning technique with our current model as well as applying our ongoing work in a real industry.

## Figures and Tables

**Table 1:** WER Performance comparison using single stream and multi-stream input.

Input	Dev	Test
RGB	25.4	26.6
Keypoint	37	36.7
**RGB + Keypoint**	**24.0**	**24.3**

**Table 2:** WER Performance comparison using different attention configuration.

Configuration	Dev	Test
Spatial + Early Temporal + Late Temporal Attention	45.2	45.2
Early Temporal + Late Temporal Attention	43.9	43.6
Spatial Attention	24.7	24.3
Early Temporal Attention	23.8	23.9
**Late Temporal Attention**	**20.5**	**21.5**

**Table 3:** WER Performance comparison using different late temporal attention configuration.

Configuration	Dev	Test
Late Temporal Attention without pooling	30.4	31.2
Late Temporal Attention with pooling	**20.5**	**21.5**

**Table 4:** WER Performance comparison using different sequence learning configuration.

Configuration	Dev	Test
Sequence Learning using LSTM	30.4	31.2
Sequence Learning using Bi-LSTM	**20.5**	**21.5**

**Table 5:** WER Performance comparison on the CSL dataset.

Methods	Test
LS-HAN [41]	17.3
DenseTCN [48]	14.3
CTF [49]	11.2
SubUNet [35]	11.0
HLSTM [44]	10.2
SF-Net [50]	3.8
FCN [51]	3.0
STMC [17]	2.1
VAC [23]	1.6
Ours (STMF)	0.8
**Ours (STAMF)**	**0.7**

**Table 6:** WER Performance comparison on the RWTH-PHOENIX dataset.

Methods	Dev	Test
CMLLR [33]	55.0	53.0
1 Million Hands [52]	47.1	45.1
SubUNet [35]	40.8	40.7
Hybrid CNN-HMM [6]	31.6	32.5
SAN [53]	29.0	29.7
SFL [54]	26.2	26.8
CNN + LSTM + HMM [34]	26.0	26.0
DNF [55]	23.8	24.4
FCN [51]	23.7	23.9
CMA [56]	21.3	21.9
VAC [23]	21.2	22.3
**STMC [17]**	21.1	**20.7**
Ours (STMF)	24.0	24.3
**Ours (STAMF)**	**20.5**	21.5
